# Adult Pancreatoblastoma: An Uncommon Pancreatic Malignancy

**DOI:** 10.7759/cureus.48063

**Published:** 2023-10-31

**Authors:** Aroosh Hussain, Jawaad Farrukh

**Affiliations:** 1 Radiology, Royal Stoke University Hospital, Stoke-On-Trent, GBR

**Keywords:** abdominal pain differential diagnosis, paediatric pancreatic neoplasm, pancreatic neoplasms, pancreatic malignancy, pancreatoblastoma

## Abstract

In this report we present a rare case of pancreatoblastoma in an adult patient. Whilst they are amongst the most common malignant pancreatic tumours in children, presentations in adults are exceedingly rare, with a small number of reported cases. Its presentation is often non-specific in terms of clinical examination, and subsequent imaging can show similar findings to those seen in benign neoplasms. This report highlights the difficulty of achieving a diagnosis and subsequent treatment of such an uncommon disease. Biopsy and resultant histology are essential in diagnosis and surgical resection remains the preferred modality of treatment. However, the use of chemotherapy and its efficacy in adults remains unclear, and the prognosis documented in existing literature for adults is worse when compared to paediatric presentations. This case emphasises the need to consider pancreatoblastoma as a differential diagnosis when suspecting pancreatic or abdominal malignancies to achieve early detection and diagnosis, in order to provide optimal treatment and improve patient outcomes.

## Introduction

Pancreatoblastoma is a rare type of malignant pancreatic tumour more commonly found in childhood in comparison to adults [1,2]. They form 25% of pancreatic neoplasm cases that are diagnosed in the first decade of life [3]. This makes them one of the most common malignant pancreatic tumours in children, with the mean age of diagnosis being four years [3,4]. With pancreatoblastomas making up less than 1% of pancreatic neoplasms, adult cases are exceedingly rare; to date only 74 cases have been reported in literature [4]. Its presentation is often varied and non-specific, with the most common presenting complaint being abdominal pain [5]. There are no symptoms specific to pancreatoblastoma and other signs include weight loss, abdominal masses, diarrhoea and less commonly jaundice [3,6,7]. Imaging can also present with similar findings to those seen in malignant and benign neoplasms [8]. Tumour markers such as alpha-fetoprotein (AFP), carcinoembryonic antigen (CEA) and Ca 19-9 and are often normal in adults, and whilst elevated AFP and Ca 19-9 can occur, it is rare [4,6,8]. The often non-specific presentation of the disease in conjunction with the fact that tumour markers are not likely to contribute to a diagnosis can make pancreatoblastoma a difficult diagnosis to achieve without biopsy and subsequent histology.

This case report presents a rare example of a pancreatoblastoma presenting in an adult and demonstrates the difficulty of diagnosing such a malignancy, as well as the challenges of treating this disease. There remains a lack of universal guidance in the management of pancreatoblastoma in adults. Whilst surgical resection remains the primary treatment of choice where possible, the role of adjuvant chemotherapy and which regimens to use still needs to be firmly established.

## Case presentation

**Figure 1:**
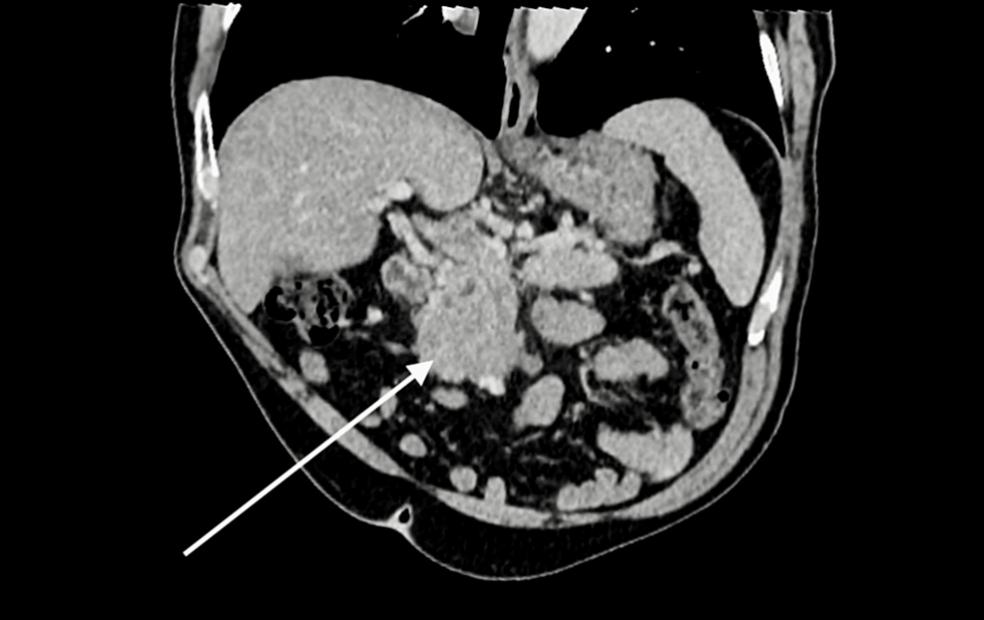
Contrast-enhanced reformatted abdominopelvic Computerized Tomography (CT) scan demonstrates interval growth of a pancreatic head soft tissue mass after two years.

**Figure 2:**
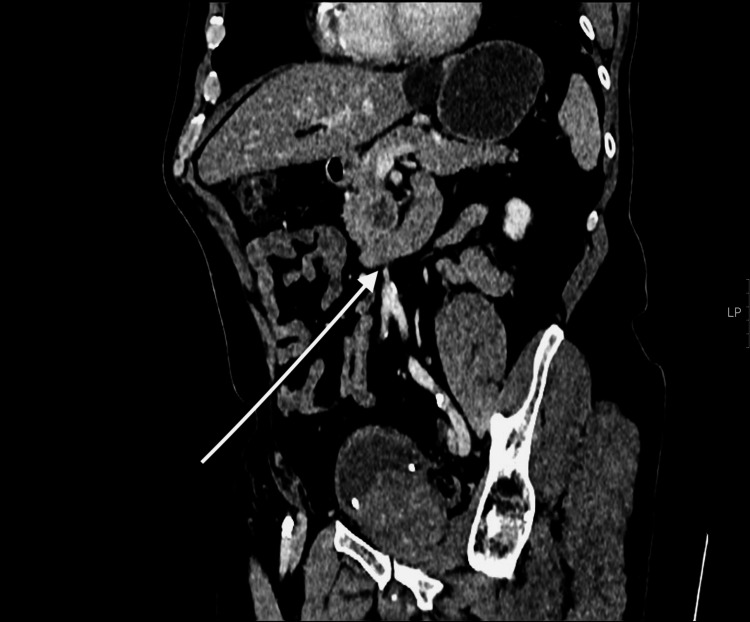
Contrast-enhanced reformatted abdominopelvic Computerized Tomography (CT) scan demonstrates well-circumscribed pancreatic head lesion.

A 75-year-old male with an Eastern Cooperative Oncology Group (ECOG) performance status score of 0 presented to the Emergency Department in September of 2022 with chest pain that was also associated with abdominal pain and nausea, these symptoms began in August of 2022. His observations and routine bloods were unremarkable at the time of this presentation, and his symptoms were thought to be due to the patient’s known history of irritable bowel syndrome. The patient also had a past medical history of hypertension and benign prostatic hyperplasia. These symptoms persisted and he presented once again in November of 2022 with intermittent chest pain and abdominal pain, as well as a reduction in appetite and weight loss. Routine blood tests and observations were once again normal, and subsequent CEA and Ca 19-9 testing were also unremarkable. The patient was then listed for a gastroscopy given his persistent symptoms, which did not show any sign of malignancy. A subsequent Computerized Tomography (CT) scan (Figure 1) showed a significant increase in size of a known pancreatic head mass (Figure 2), from 2.8cm to 6 x 6.5cm with infiltrates into the duodenum and areas of the small bowel. The mass also showed partial encapsulation of the superior mesenteric artery (SMA) and a new focal hepatic lesion, raising suspicion of metastasis.

Further magnetic resonance imaging (MRI) imaging confirmed the size and location of the disease, as well as extensive superior mesenteric vein (SMV) and portal vein involvement with solitary liver metastases. Subsequent endoscopic ultrasound (EUS) guided core biopsy confirmed pancreatoblastoma. Since the mass was locally advanced and involved several blood vessels it was deemed inoperable due to the complexity of its location and the concern of liver metastases.

The patient was offered a FOLFIRINOX regimen of chemotherapy and managed to complete one cycle before complications arose, and a subsequent admission occurred in March 2023. An interim CT scan during this period showed local disease progression which resulted in a malignant stricture of the common bile duct (CBD), this led to a biliary stent insertion due to CBD obstruction caused by the increased size of the malignancy. The CT scan also showed significant progression in the size of the pancreatic head tumour and the size of the liver metastases despite one cycle of chemotherapy.

Following the patient's recovery from his endoscopic retrograde cholangiopancreatography (ERCP) and stent insertion, the second cycle of FOLFIRINOX was attempted and unfortunately suspended as the patient could no longer tolerate the treatment due to side effects such as poor appetite, nausea and vomiting, and excessive weight loss. A decision was then made in conjunction with the patient that he would be for best supportive care. Five months after this decision was made the patient remains stable and currently has palliative care input in the community from his local hospice to manage his symptoms.

## Discussion

Pancreatoblastomas are slow-growing tumours that originate from the exocrine cells of the pancreas. They frequently present in a non-specific manner and given their slow growth by the time patients often present, the tumours can grow to a significant size. As a result, symptoms such as abdominal pain or distension can often be the primary presenting complaint [9]. Whilst less common in adults they have predominantly been shown to have poorer outcomes in comparison to paediatric cases [3]. Cavallini et al. highlight an average prognosis of 18 months in adults [1].

This report highlights the presence of a pancreatic head tumour, which is the most common site for a pancreatoblastoma. Despite this, it can be found at any point of the pancreas, as case reports have shown findings of tumours both in the body and tail also [4]. Due to the nature of their slow insidious growth and often late presentation, pancreatoblastomas can commonly present with metastases, this has previously been shown to occur in 25% of cases, with up to 59% of cases being shown to develop metastases both before and after diagnosis [4,6]. In keeping with its propensity for local infiltration, the most common site for metastases is the liver which also occurred in our report [4,5].

Due to the rarity of this disease, much remains unknown about its aetiology with links being made with syndromes such as familial adenomatous polyposis (FAP) or Beckwith-Wiedemann syndrome [10]. Achieving a diagnosis purely on radiographic signs is difficult as most pancreatoblastomas present as large, well-defined, heterogenous masses. Enhancement can often be shown on contrast-enhanced CT scans as well as MRI [4,6]. Given that the majority of radiographic features of pancreatoblastomas are non-specific they can be mistaken for more common neoplasms such as neuroendocrine tumours or adenocarcinomas [6]. The difficulty in providing an initial diagnosis means that tumour biopsy and subsequent histology are therefore key for diagnosis.

Surgical resection is the main and most effective treatment modality. However, this is dependent on several factors, such as the progression of the disease, its location, and the presence of metastases. Resection can result in a distal pancreatectomy including a splenectomy, depending on whether lesions are present on the body and tail. Prognosis can be positive with a complete resection of the tumour, as surgery has been shown to be associated with a survival time of 15 months in comparison to just five months in patients who had unresected tumours [6,11]. Survival times were 20.5 months when both surgical resection and chemoradiotherapy were used compared to the 15 months shown in patients who were only treated with surgical resection [11].

Whilst chemotherapy has been shown to have a role in both pre- and post-surgery there remains a distinct absence of universal treatment guidelines for adults in this area. Chemotherapy plays a significant role, particularly when the disease is unresectable, involves local blood vessels, or has metastasised, all of which occurred in our case. Although chemotherapy was given and unfortunately not tolerated in this case, at present there remains no clear optimal regimen. Despite this chemotherapy does appear to be of some benefit in palliating metastatic or recurrent disease [11].

## Conclusions

Whilst pancreatoblastoma is a rare malignancy especially in adults, given its non-specific presentation both on clinical and imaging examination it can be very difficult to diagnose without a successful biopsy. Therefore, it must be considered as a differential diagnosis when suspecting atypical pancreatic malignancies, in order to achieve as early detection and diagnosis as possible. Early detection is critical, as often patients will present once the tumour has grown to a substantial size and achieved a significant degree of local infiltration. This can pose a challenging scenario for surgeons, as was shown in this case with the malignancy being deemed inoperable.

There remains a lack of an optimal standardised treatment regimen in adults due to the rarity of this disease highlighting the need for further research and analysis. This case emphasises the importance of early detection and diagnosis, as they play a key role in maximising the possibility of surgical resection as a treatment option and ultimately improving patient outcomes. 

## References

[1] Cavallini A, Falconi M, Bortesi L, Crippa S, Barugola G, Butturini G (2009). Pancreatoblastoma in adults: a review of the literature. Pancreatology.

[2] Glick RD, Pashankar FD, Pappo A, Laquaglia MP (2012). Management of pancreatoblastoma in children and young adults. J Pediatr Hematol Oncol.

[3] Klimstra DS, Wenig BM, Adair CF, Heffess CS (1995). Pancreatoblastoma. A clinicopathologic study and review of the literature. Am J Surg Pathol.

[4] Omiyale AO (2021). Adult pancreatoblastoma: current concepts in pathology. World J Gastroenterol.

[5] Omiyale AO (2015). Clinicopathological review of pancreatoblastoma in adults. Gland Surg.

[6] Zouros E, Manatakis DK, Delis SG, Agalianos C, Triantopoulou C, Dervenis C (2015). Adult pancreatoblastoma: a case report and review of the literature. Oncol Lett.

[7] Patterson KN, Trout AT, Shenoy A, Abu-El-Haija M, Nathan JD (2022). Solid pancreatic masses in children: a review of current evidence and clinical challenges. Front Pediatr.

[8] Li J, Peng C, Fan X, Wang L, Wang J (2021). Adult pancreatoblastoma: a case report. J Int Med Res.

[9] Morrissey G, Cohen P, Julve M (2020). Rare case of adult pancreatoblastoma. BMJ Case Rep.

[10] Rojas A, Wodskow J, Hogg ME (2023). Adult pancreatoblastoma: a rare pancreatic tumor. J Gastrointest Surg.

[11] Salman B, Brat G, Yoon YS (2013). The diagnosis and surgical treatment of pancreatoblastoma in adults: a case series and review of the literature. J Gastrointest Surg.

